# Mannan oligosaccharides alleviate oxidative injury in the head kidney and spleen in grass carp (*Ctenopharyngodon idella*) via the Nrf2 signaling pathway after *Aeromonas hydrophila* infection

**DOI:** 10.1186/s40104-023-00844-1

**Published:** 2023-04-15

**Authors:** Zhiyuan Lu, Lin Feng, Weidan Jiang, Pei Wu, Yang Liu, Jun Jiang, Shengyao Kuang, Ling Tang, Shuwei Li, Chengbo Zhong, Xiaoqiu Zhou

**Affiliations:** 1 Animal Nutrition Institute, Sichuan Agricultural University, Chengdu, 611130 China; 2 Fish Nutrition and Safety Production University Key Laboratory of Sichuan Province, Sichuan Agricultural University, Chengdu, 611130 China; 3 Key Laboratory for Animal Disease-Resistance Nutrition of China Ministry of Education, Sichuan Agricultural University, Chengdu, 611130 China; 4 Sichuan Animal Science Academy, Sichuan Animtech Feed Co. Ltd, Chengdu, 610066 China; 5 Animal Breeding and Genetics Key Laboratory of Sichuan Province, Animal Nutrition Institute, Sichuan Academy of Animal Science, Chengdu, 610066 China

**Keywords:** Antioxidant, Apoptosis, Functional organs, Grass carp, Mannan oligosaccharides

## Abstract

**Background:**

Mannan oligosaccharides (MOS) are recommended as aquaculture additives owing to their excellent antioxidant properties. In the present study, we examined the effects of dietary MOS on the head kidney and spleen of grass carp (*Ctenopharyngodon idella*) with *Aeromonas hydrophila* infection.

**Methods:**

A total of 540 grass carp were used for the study. They were administered six gradient dosages of the MOS diet (0, 200, 400, 600, 800, and 1,000 mg/kg) for 60 d. Subsequently, we performed a 14-day *Aeromonas hydrophila* challenge experiment. The antioxidant capacity of the head kidney and spleen were examined using spectrophotometry, DNA fragmentation, qRT-PCR, and Western blotting.

**Results:**

After infection with *Aeromonas hydrophila*, 400–600 mg/kg MOS supplementation decreased the levels of reactive oxygen species, protein carbonyl, and malonaldehyde and increased the levels of anti-superoxide anion, anti-hydroxyl radical, and glutathione in the head kidney and spleen of grass carp. The activities of copper-zinc superoxide dismutase, manganese superoxide dismutase, catalase, glutathione *S*-transferase, glutathione reductase, and glutathione peroxidase were also enhanced by supplementation with 400–600 mg/kg MOS. Furthermore, the expression of most antioxidant enzymes and their corresponding genes increased significantly with supplementation of 200–800 mg/kg MOS. mRNA and protein levels of nuclear factor erythroid 2-related factor 2 also increased following supplementation with 400–600 mg/kg MOS. In addition, supplementation with 400–600 mg/kg MOS reduced excessive apoptosis by inhibiting the death receptor pathway and mitochondrial pathway processes.

**Conclusions:**

Based on the quadratic regression analysis of the above biomarkers (reactive oxygen species, malondialdehyde, and protein carbonyl) of oxidative damage in the head kidney and spleen of on-growing grass carp, the recommended MOS supplementation is 575.21, 557.58, 531.86, 597.35, 570.16, and 553.80 mg/kg, respectively. Collectively, MOS supplementation could alleviate oxidative injury in the head kidney and spleen of grass carp infected with *Aeromonas hydrophila*.

**Supplementary Information:**

The online version contains supplementary material available at 10.1186/s40104-023-00844-1.

## Background

Intensive farming has become the most crucial mode of production for aquaculture because of its high efficiency [1]. However, intensive farming may pose serious problems. High-density fish farming is associated with excessive oxidative stress, decreased disease resistance, and increased disease incidence [2–4]. Thus, developing a reliable method to reduce excessive oxidative stress in farmed fish is essential. The head kidney and spleen play a vital role in the management of oxidative stress in fish [5, 6]. Dynamic changes in antioxidant systems are primarily associated with oxidative stress responses [7]. Mannan oligosaccharides (MOS) improve fish health and growth; therefore, supplementing fish feed with MOS is a potential measure to avoid oxidative stress [8]. However, whether MOS affect the antioxidant systems of the head kidney and spleen remains unknown. Studies on the effects of MOS on the head kidney and spleen have focused on phenotypic indicators [9–11]. Therefore, a systematic and thorough study is necessary to determine whether MOS supplementation affects the antioxidant system of the head kidney and spleen in fish.

Reactive oxygen species (ROS) cause oxidative stress. ROS, such as superoxide, hydrogen peroxide, and singlet oxygen, can interact with other signaling molecules [12]. After bacterial infection, fish serum ROS levels increase as a systemic stress response [7, 13, 14]. However, excessive ROS cause oxidative damage to intracellular proteins and lipids. Protein carbonyl (PC) levels are used as an index of the extent of oxidative damage to proteins, whereas malondialdehyde (MDA) levels are used as markers for lipid oxidation [15]. Dietary MOS supplementation improved phagocytic activity in the head kidney of European sea bass (*Dicentrarchus labrax*) [9], boosted respiratory burst in the head kidney of juvenile hybrid groupers (*Epinephelus lanceolatus*♂ × *Epinephelus fuscoguttatus*♀) [11], and decreased inducible nitric oxide synthase (iNOS) gene expression in the head kidney and spleen of greater amberjack (*Seriola dumerili Risso* 1810) juveniles [10]. Changes in phagocytic activity, respiratory burst, and iNOS expression are accompanied by ROS production [16, 17]. However, these studies did not investigate the levels of ROS and oxidative damage markers MDA and PC, nor did they design tests to induce an oxidative stress response. We previously reported that MOS could reduce the levels of ROS and oxidative damage products, PC and MDA, in the intestine during *Aeromonas hydrophila* infection [18]. However, the cell type composition and structure of different organs in fish are different; thus, they may exhibit a different response to bacterial infection [19]. Therefore, investigating the effects of MOS on the oxidative stress response and biomarkers in the head kidney and spleen during *A. hydrophila* infection is crucial.

Fish have evolved an efficient antioxidant system to prevent ROS accumulation and repair oxidative damage [20]. For example, antioxidant enzymes such as superoxide dismutase (SOD), catalase (CAT), glutathione *S*-transferase (GST), glutathione reductase (GR), and glutathione peroxidase (GPx), scavenge excessive free radicals mainly through synergistic interaction [21]. Glutathione is one of the major components of the nonenzymatic antioxidant defense system and can inhibit free radical production by acting as an electron donor [21]. To date, no study has been conducted to determine whether MOS affects antioxidant enzymes and non-enzymatic antioxidants in the head kidney and spleen. To the best of our knowledge, the intestinal flora produces short-chain fatty acids (SCFAs) (e.g., acetate, propionate, and butyrate) from prebiotics through a multidisciplinary fermentation process [22–25]. We previously confirmed that MOS could significantly increase intestinal butyrate content [18]. A study in broiler chicks showed that butyrate could significantly increase the activity of SOD in the serum and blood [26]. The expression of most antioxidant enzymes is regulated by *Nrf2* [27]. However, we lack reports on the regulation of *Nrf2* by MOS in the head kidney and spleen. According to a study on piglets, MOS supplementation may enhance phosphorus absorption in the intestine [28]. Phosphorus can promote *Nrf2* expression in the head kidney and spleen of grass carp [29]. Therefore, we hypothesize that the regulation of *Nrf2* signaling by MOS in the head kidney and spleen of fish may be achieved by increasing the apparent digestibility of phosphorus. However, the specific regulatory mechanisms remain unclear and require further investigation.

According to FAO statistics (FAO, 2020) [30], grass carp is a freshwater fish with the highest global cultivation and has tremendous economic value. It can be a source of high-quality protein for humans. Thus, we aimed to comprehensively investigate the effects of MOS supplementation on the antioxidant stress response of grass carp immune organs in an actual production environment based on the infection of pathogenic microorganisms. Further, we aimed to determine the optimal MOS dosage based on oxidative stress biomarkers of functional organs and to elucidate how MOS positively affects the head kidney and spleen. We speculate that dietary MOS can effectively alleviate the oxidative damage of head kidney and spleen of grass carp caused by pathogen infection. To confirm this hypothesis, we investigated typical biomarkers of oxidative stress, phenotypic indicators of antioxidant enzymes, expression of related genes, and protein levels of associated signaling molecules. We believe this study may serve as a practical reference for the commercial feed preparation of grass carp.

## Materials and methods

### Study design

Mannan oligosaccharides (MOS) diet (Sciphar Hi-Tech Industry, Xi’an, China, purity: 99.12%) was prepared as described in our previous study [18]. A mixture of fish meal, soybean protein concentrate, and gelatin was used as the dietary protein source. Fish oil and soybean oil were used as dietary lipid sources. A summary of the formulation and proximate composition analysis of the experimental diets is shown in Table 1. MOS was added to the basal diet at six concentrations (0, 200, 400, 600, 800, and 1,000 mg/kg) to replace corn starch (standard carbohydrates). This dose was selected based on the appropriate levels of MOS required to promote the growth performance of other cyprinid fish in freshwater, such as allogynogenetic crucian carp (240–480 mg/kg) [32], and similar results were obtained in common carp (1,000–1,500 mg/kg) [33] and blunt snout bream (200–400 mg/kg) [34].

**Table 1:** Composition and nutrient content of the diet (dry matter basis), %

Ingredients	Content, %	Nutrient	Content, %
Fish meal	7.80	Crude protein^d^	28.69
Gelatin	6.00	Crude lipid^d^	5.36
Soybean protein concentrated	26.00	n-3^d^	1.04
Corn starch	19.90	n-6^d^	0.96
α-starch	24.00	Available phosphorus^d^	0.40
Fish oil	2.34		
Soybean oil	1.81		
Cellulose	5.00		
Ca(H_2_PO4)_2_	1.30		
Vitamin premix^a^	1.00		
Mineral premix^b^	2.00		
MOS premix^c^	1.00		
Choline chloride (50%)	1.00		
Ethoxyquin (30%)	0.05		
*DL*-Met (99%)	0.61		
*L*-Trp (99%)	0.08		
Thr (98.5%)	0.11		

^a^Per kilogram of vitamin premix (g/kg): retinyl acetate (500,000 IU/g), 0.39; cholecalciferol (500,000 IU/g), 0.40; *DL*-α-tocopherol acetate (50%), 23.23; menadione (22.9%), 0.83; cyanocobalamin (1%), 0.94; *D*-biotin (2%), 0.75; folic acid (95%), 0.42; thiamine nitrate (98%), 0.09; ascorhyl acetate (95%), 9.77; niacin (99%), 4.04; meso-inositol (98%), 19.39; calcium-*D*-pantothenate (98%), 3.85; riboflavin (80%), 0.73; pyridoxine hydrochloride (98%), 0.62. All ingredients were diluted with corn starch to 1 kg

^b^Per kilogram of mineral premix (g/kg): MnSO_4_⋅H_2_O (31.8% Mn), 2.6590; MgSO_4_⋅H_2_O (15.0% Mg), 200.0000; FeSO_4_⋅H_2_O (30.0% Fe), 12.2500; ZnSO_4_⋅H_2_O (34.5% Zn), 8.2460; CuSO_4_⋅5H_2_O (25.0% Cu), 0.9560; KI (76.9% I), 0.0650; Na_2_SeO_3_ (44.7% Se), 0.0168. All ingredients were diluted with corn starch to 1 kg

^c^MOS premix (mg/kg): premix was added to obtain graded levels of MOS

^4^Crude protein and crude lipid content were measured value. Available phosphorus, n-3 and n-6 contents calculated according to NRC (2011) [31]

### Animals and experimental management

The design of the current study and protocols used were approved by the Laboratory Animal Care and Use of Animal Nutrition Institute (LACUANI) of Sichuan Agricultural University (permit no. LZY-2018114005). Tong Wei fisheries (Sichuan, China) provided healthy grass carp. The initial average weight of the fish was 215.85 ± 0.30 g (mean ± SD). All fish were acclimatized for a month before the start of the experiment. All fish in the domestication period were fed the control diet, and the other conditions were the same as those in the formal experiment period. Subsequently, 540 fish were randomly assigned to six dietary treatment groups with three replicates per treatment group, 30 fish per replicate after acclimatization. The growth trial was conducted for 60 d, with four feedings per day. The leftover diets were collected using 100-cm mesh discs after 30 min of feeding. The water temperature stayed within a range of 28.5 ± 2.0 °C (mean ± SD), and the pH was maintained at 7.5 ± 0.3 (mean ± SD). The dissolved oxygen concentration was less than 6.0 mg/L throughout the experiment. In addition, the trial was conducted in natural light (natural long days in summer: 14 h light/10 h dark).

### Challenge test

To investigate the effects of dietary MOS supplementation on antioxidant homeostasis in fish head kidney and spleen, we performed a challenge trial at the end of the growth trial. *A. hydrophila* (FDL20120711) was provided by the Department of Veterinary Medicine of Sichuan Agricultural University. Briefly, the challenge was conducted with 15 randomly selected fish from each group. *A. hydrophila* (1.0 mL) was administered intraperitoneally to selected fish. The concentration of bacteria was 2.5 × 10^8^ colony-forming units (CFU)/mL. Normal saline [0.85% NaCl (w/v) solution] was administered to the fish in the saline group. Experimental management and environmental control of the challenge trial were consistent with those of the growth trial. The *A. hydrophila* challenge model was successfully established in our previous study [18].

### Sampling and biochemical parameters analysis

In accordance with the LACUANI regulations, anesthesia was administered to all fish using benzocaine after the challenge trial. Subsequently, the head kidney and spleen from grass carp were quickly separated and placed in liquid nitrogen for temporary storage, as described in our previous study [35]. Samples were stored at −80 °C until further use. To measure oxidative damage biomarkers (ROS, PC, and MDA) and antioxidant parameters (anti-superoxide anion (ASA), anti-hydroxyl radical (AHR), superoxide dismutase (SOD), catalase (CAT), glutathione peroxidase (GPx), glutathione *S*-transferase (GST), glutathione reductase (GR), and glutathione (GSH), head kidney and spleen samples were pre-processed prior to the use of commercial kits. Briefly, 0.5 g of head kidney or spleen tissue was weighed, and nine times the volume of normal saline was added, the tissue was cut as much as possible with small ophthalmic scissors, and then the high-throughput tissue grinder (SCIENTZ-48, SCIENTZ, Ningbo, China) was used to grind into 10% tissue homogenate (w/v), followed by centrifugation at 6,000 × *g* for 20 min at 4 °C. The supernatants were collected and stored for further analysis. Table 2 provides a list of biomarkers and related enzyme activity analysis methods used. ROS production was detected using a commercial kit purchased from Beyotime (Shanghai, China). The protein concentrations of MDA, PC, ASA, AHR, SOD, CAT, GPx, GST, GR, and GSH were determined using commercial kits (Nanjing Jiancheng Institute of Bioengineering, Nanjing, China).

**Table 2:** The analysis method of biomarker and enzymes activity related parameters

Indices (commercial kit)	Method and principle of determination	Reference codes
Reactive oxygen species (ROS)	2,7-dichlorofluorescin-diacetate (DCFH-DA) method, reactive oxygen species can oxidize non-fluorescent DCFH to produce fluorescent DCF, and the absorbance was assessed at the characteristic absorption peak.	S0033M
Malondialdehyde (MDA)	Thibabituric acid method, MDA can be condensed with thibabituric acid to form a red product and the absorbance was assessed at the characteristic absorption peak.	A003–1-2
Protein carbonyl (PC)	The 2,4-dinitrophenylhydrazine (DNPH) method, the carbonyl group reacts with DNPH to form a red product, and the absorbance was assessed at the characteristic absorption peak.	A087–1-2
Anti-superoxide anion (ASA)	Spectrophotometric method, superoxide anion free radical, electron transfer substance and chromogenic agent react together to show purplish red, and the absorbance was assessed at the characteristic absorption peak.	A052–1-1
Anti-hydroxy radical (AHR)	Fenton reaction method, the Fenton reaction produces hydroxyl radicals, which can react with griess reagent to produce red products after combining with electron acceptor, and the absorbance was assessed at the characteristic absorption peak.	A018–1-1
Superoxide dismutase (SOD)	Hydroxylamine method, the reaction system of xanthine and xanthine oxidase produces superoxide anion free radicals, which oxidize hydroxylamine to form nitrite, and show purplish red products under the action of chromogenic agents, and the absorbance was assessed at the characteristic absorption peak.	A001–2
Catalase (CAT)	Ammonium molybdate method, the decomposition of H_2_O_2_ by catalase can be halted by the addition of ammonium molybbate, and the remaining hydrogen peroxide acts with ammonium molybbate to produce a yellow complex, and the absorbance was assessed at the characteristic absorption peak.	A007–1-1
Glutathione peroxidase (GPx)	Spectrophotometric method, GPx can promote the reaction of H_2_O_2_ with reduced glutathione (GSH) to form H_2_O and oxidized glutathione (GSSG), GSH reacts with dinitrobenzoic acid to form 5-thio-dinitrobenzoic acid anion, which shows a stable yellow color, and the absorbance was assessed at the characteristic absorption peak.	A005–1-2
Glutathione-S-transferase (GST)	Spectrophotometric method, GST has the ability to catalyse the binding of reduced GSH to 1-chloro-2,4-dinitrobenzene (CDNB substrate). Within a certain reaction time, the activity of GST is linearly related to the change of substrate concentration before and after the reaction, and the absorbance was assessed at the characteristic absorption peak.	A004–1-1
Glutathione reductase (GR)	Ultraviolet spectrophotometry method, when GSSG is catalyzed by glutathione reductase GR and supplied with hydrogen by NADPH, GSSG is reduced to reduced GSH, GSH increases and NADPH decreases, and the absorbance was assessed at the characteristic absorption peak.	A062–1-1
Glutathione (GSH)	Spectrophotometric method, Reduced GSH can react with dithio-dinitrobenzoic acid (DTNB) to form a yellow compound, and the absorbance was assessed at the characteristic absorption peak.	A006–2-1
Protein concentrations	Coomassie brilliant blue method, Protein molecules have -NH_3_^+^ groups, and when the brown-red Coomassie brilliant blue chromogenic agent is added to the protein standard solution or sample, the anion on the Coomassie brilliant blue dye binds to the protein-NH_3_^+^, turning the solution blue, and the absorbance was assessed at the characteristic absorption peak.	A045–2-2

### DNA fragment analysis

Fragmented DNA was isolated from the head kidney and spleen following previously described methods [36]. Extracted DNA was analyzed on a 2% agarose gel to verify fragmentation, according to the manufacturer’s instructions. Samples were electrophoresed for 90 min at 80 V. The separated DNA fragments were visualized using Gene Genius (Syngene, Frederick, MD, USA).

### Real-time RT-PCR

Total RNA was isolated from the head kidney and spleen samples using the RNAiso Plus kit (Takara, Dalian, China), and the quality of the RNA was assessed on 1% agarose gels and Nanodrop 2000 (Thermo Fischer Scientific, Waltham, USA). Reverse transcription of head kidney and spleen RNA was performed using the PrimeScript™ RT reagent kit (Takara, Dalian, China). Primers for qRT-PCR were designed in accordance with the cloned sequences from our laboratory (Additional file 1: Table S1). After screening four internal reference genes, *β-Actin* and *GAPDH* were selected as reference genes as previously described [18, 37]. Melting curves were plotted according to the manufacturer’s instructions, and the amplification efficiency was calculated. The relative transcription levels were determined using the 2^−ΔΔCT^ method [38].

### Western blotting

Preparation of head kidney and spleen homogenates, primary and secondary antibodies, and image analysis for Western blotting were performed as described previously [18, 39]. RIPA buffer and BCA assay kits (Beyotime Institute of Biotechnology, Shanghai, China) were used for the extraction and determination of total protein in the tissues, respectively. The samples were separated using SDS-PAGE (10%) and transferred to polyvinylidene difluoride (PVDF) membranes after preparation (40 μg/lane). Primary antibodies were incubated with membranes overnight (14 h, 4 °C). After washing, the membranes were incubated with secondary antibodies at room temperature (25 ± 2.0 °C) for 90 min. We then visualized and quantified the separated proteins (NIH Image J, 1.42*q*), as described previously [18, 40]. Additional file 2: Table S2 provides detailed information on all antibodies used in the current study.

### Statistical analysis

The Shapiro–Wilk test and Levene’s test were performed before statistical analysis to determine normality and homogeneity of variance, respectively. Data were analyzed using one-way analysis of variance (ANOVA) and Duncan’s multiple comparisons at *P* < 0.05, using SPSS 27.0 (IBM Corp., Armonk, NY, USA). An orthogonal polynomial contrast method was used to test the linear and quadratic effects of the increasing number of MOS applications. Correlation analysis was performed using Pearson’s correlation test (CORR). Data visualization was performed using GraphPad 8.0 (GraphPad Software, Inc., San Diego, CA, USA) and R (v4.0.2), as well as Hiplot online tools.

## Results

### Growth performance

After the growth trial, MOS supplementation exhibited significant growth-promoting effects as indicated by the final body weight of fish and their feed intake. Compared with the control group, the final body weight and feed intake of fish were highest (mean ± SD) in fish administered 400 mg/kg MOS. Groups 1–6 were administered the following doses of MOS; 825.24 ± 2.45, 853.77 ± 11.74, 890.83 ± 2.26, 858.10 ± 11.37, 824.53 ± 5.74 and 782.63 ± 7.48, *P*_*quadratic*_ = 0.051, *F* = 9.378, *df* = 5; 702.56 ± 9.36, 760.55 ± 11.42, 854.22 ± 6.05, 769.95 ± 12.27, 706.73 ± 8.85, 638.78 ± 6.87, *P*_*quadratic*_ = 0.017, *F* = 20.758, *df* = 5, respectively.

### Biomarkers of oxidative damage

**Fig. 1:**
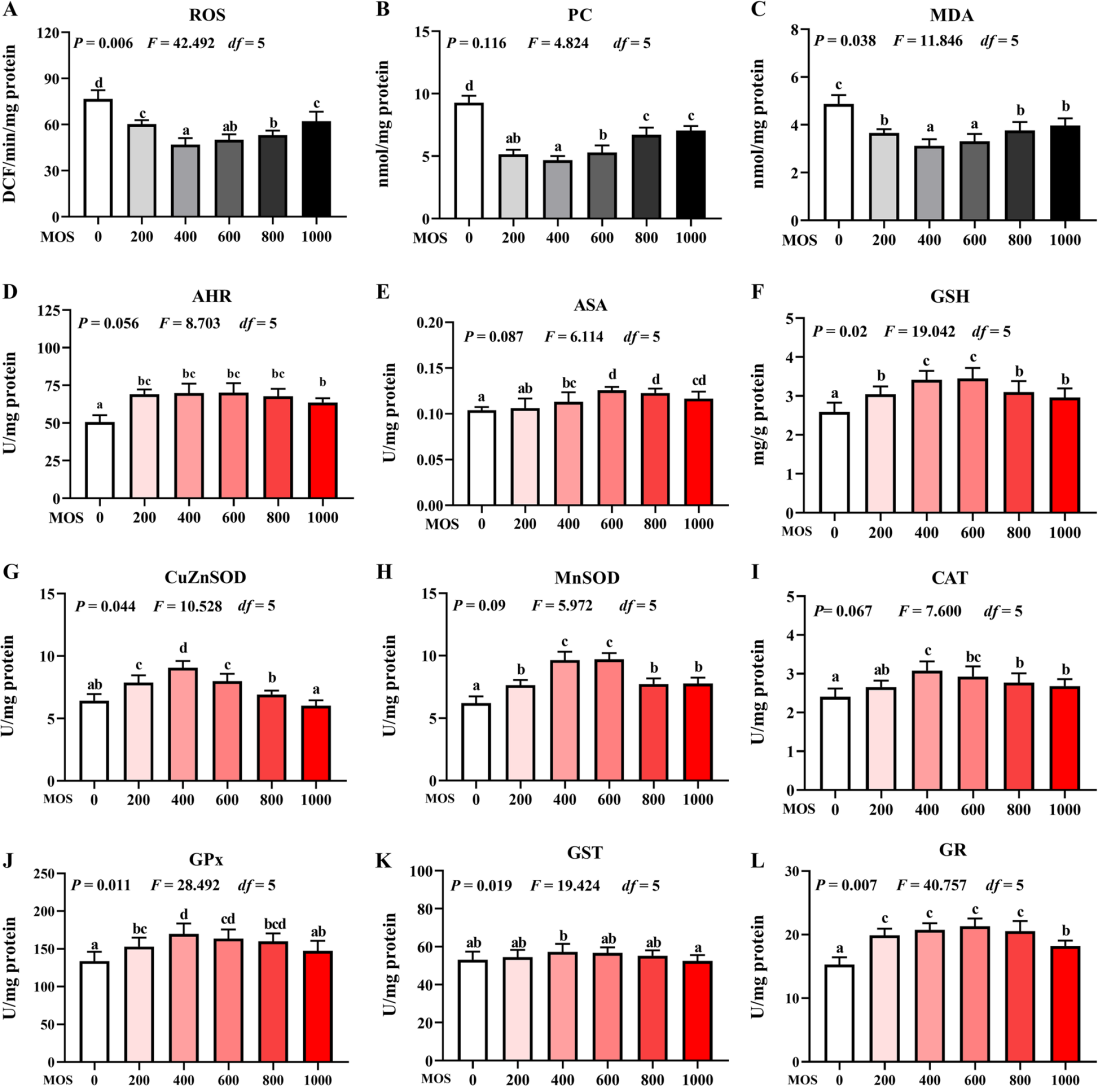
Effect of dietary MOS supplementation on antioxidant capacity in the head kidney of grass carp after infection of *Aeromonas hydrophila*. **A**–**C**, biomarkers of oxidative damage, ROS: reactive oxygen species, DCF/min/florescence; PC: protein carbonyl, nmol/mg protein; MDA: malondialdehyde, nmol/mg protein. **D**–**L**, antioxidant-related parameters, ASA: anti-superoxide anion, U/mg protein; AHR: anti-hydroxy radical, U/mg protein; CuZnSOD: copper/zinc superoxide dismutase, U/mg protein; MnSOD: manganese superoxide dismutase, U/mg protein; CAT: catalase, U/mg protein; GPx: glutathione peroxidase, U/mg protein; GST: glutathione reductase, U/mg protein; GR: glutathione reductase, U/mg protein; GSH: glutathione, nmol/mg protein. *n* = 6 for each MOS level, different letters above bars indicate significant differences

**Fig. 2:**
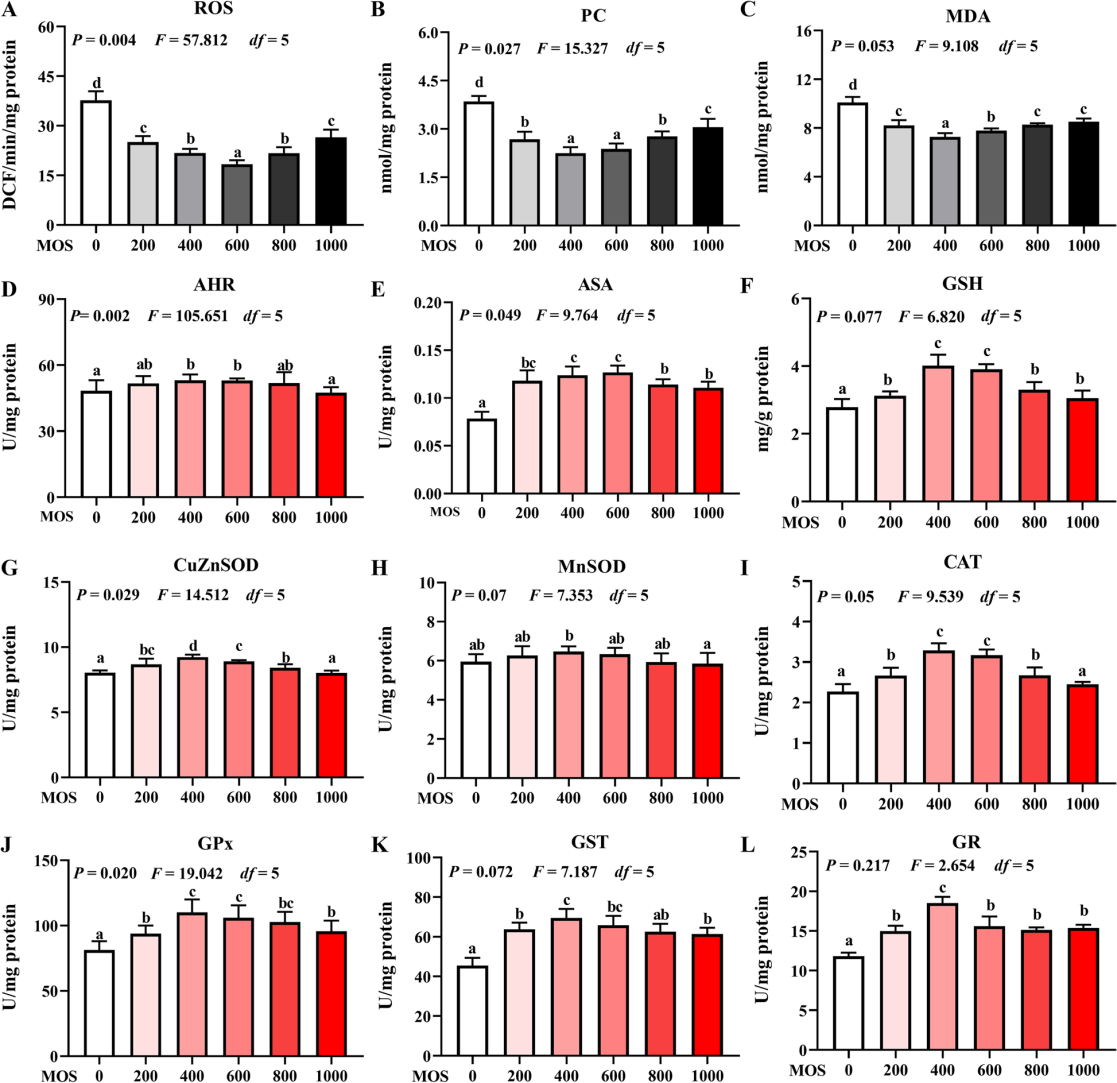
Effect of dietary MOS supplementation on antioxidant capacity in the spleen of grass carp after infection of *Aeromonas hydrophila*. **A**–**C**, biomarkers of oxidative damage, ROS: reactive oxygen species, DCF/min/florescence; PC: protein carbonyl, nmol/mg protein; MDA: malondialdehyde, nmol/mg protein. **D**–**L**, antioxidant-related parameters, ASA: anti-superoxide anion, U/mg protein; AHR: anti-hydroxy radical, U/mg protein; CuZnSOD: copper/zinc superoxide dismutase, U/mg protein; MnSOD: manganese superoxide dismutase, U/mg protein; CAT: catalase, U/mg protein; GPx: glutathione peroxidase, U/mg protein; GST: glutathione reductase, U/mg protein; GR: glutathione reductase, U/mg protein; GSH: glutathione, nmol/mg protein. *n* = 6 for each MOS level, different letters above bars indicate significant differences

To examine the effect of MOS on oxidative damage of the head kidney and spleen after *A. hydrophila* infection, levels of ROS, MDA, and PC were quantified, as shown in Fig. 1 and 2. Compared with those of the control, the ROS (*P*_*quadratic*_ = 0.006, *F* = 42.492, *df* = 5), PC (*P*_*quadratic*_ = 0.116, *F* = 4.824, *df* = 5), and MDA (*P*_*quadratic*_ = 0.038, *F* = 11.846, *df* = 5) content of the head kidney significantly decreased with MOS supplementation and attained their lowest levels upon treatment with 400 mg/kg MOS, followed by a gradual increase in their levels with an increase in MOS concentration. ROS (*P*_*quadratic*_ = 0.004, *F* = 57.812, *df* = 5), PC (*P*_*quadratic*_ = 0.027, *F* = 15.327, *df* = 5), and MDA (*P*_*quadratic*_ = 0.053, *F* = 9.108, *df* = 5) levels in the spleen also significantly decreased with MOS supplementation and reached the lowest levels at 600, 400, and 400 mg/kg of MOS, respectively. The anti-superoxide anion (ASA) (*P*_*quadratic*_ = 0.087, *F* = 6.114, *df* = 5) and anti-hydroxyl radical (AHR) (*P*_*quadratic*_ = 0.056, *F* = 8.703, *df* = 5) content of the head kidney significantly increased with MOS supplementation. It peaked at 600 and 400 mg/kg and then gradually decreased with an increase in MOS concentration. The ASA content (*P*_*quadratic*_ = 0.049, *F* = 9.764, *df* = 5) of the spleen significantly increased with MOS supplementation and reached the highest level at 600 mg/kg of MOS; AHR content (*P*_*quadratic*_ = 0.002, *F* = 105.651, *df* = 5) increased from 0 to 600 mg/kg, and then its level gradually decreased with increase in MOS concentration.

### Biochemical analysis of antioxidants

After infection with *A. hydrophila*, the effect of MOS supplementation on antioxidant capacities in the head kidney and spleen were examined; further, its effect on activities of antioxidant enzymes and GSH levels was determined (Fig. 1 and 2). The activities of CuZnSOD (*P*_*quadratic*_ = 0.044, *F* = 10.528, *df* = 5), MnSOD (*P*_*quadratic*_ = 0.090, *F* = 5.972, *df* = 5), CAT (*P*_*quadratic*_ = 0.067, *F* = 7.600, *df* = 5), GPx (*P*_*quadratic*_ = 0.011, *F* = 28.492, *df* = 5), GST (*P*_*quadratic*_ = 0.019, *F* = 19.424, *df* = 5), GR (*P*_*quadratic*_ = 0.007, *F* = 40.757, *df* = 5), and GSH (*P*_*quadratic*_ = 0.020, *F* = 19.042, *df* = 5) in the head kidney significantly increased upon MOS supplementation and reached the highest levels at 400, 600, 400, 400, 400, 600, and 600 mg/kg, respectively, followed by a gradual decrease with further increase in MOS concentration compared with the activities of these enzymes in the head kidney of animals from the control group. The activities of CuZnSOD (*P*_*quadratic*_ = 0.029, *F* = 14.512, *df* = 5), MnSOD (*P*_*quadratic*_ = 0.070, *F* = 7.353, *df* = 5), CAT (*P*_*quadratic*_ = 0.050, *F* = 9.539, *df* = 5), GPx (*P*_*quadratic*_ = 0.020, *F* = 19.042, *df* = 5), GST (*P*_*quadratic*_ = 0.072, *F* = 7.187, *df* = 5), GR (*P*_*quadratic*_ = 0.217, *F* = 2.654, *df* = 5), and GSH (*P*_*quadratic*_ = 0.077, *F* = 6.820, *df* = 5) in the spleen significantly increased upon MOS supplementation and reached their highest levels at 400 mg/kg, followed by a gradual decrease with increase in MOS concentration, as compared with the activities of these enzymes in the spleen of animals from the control group.

### Expression of genes related to antioxidant enzyme

**Fig. 3:**
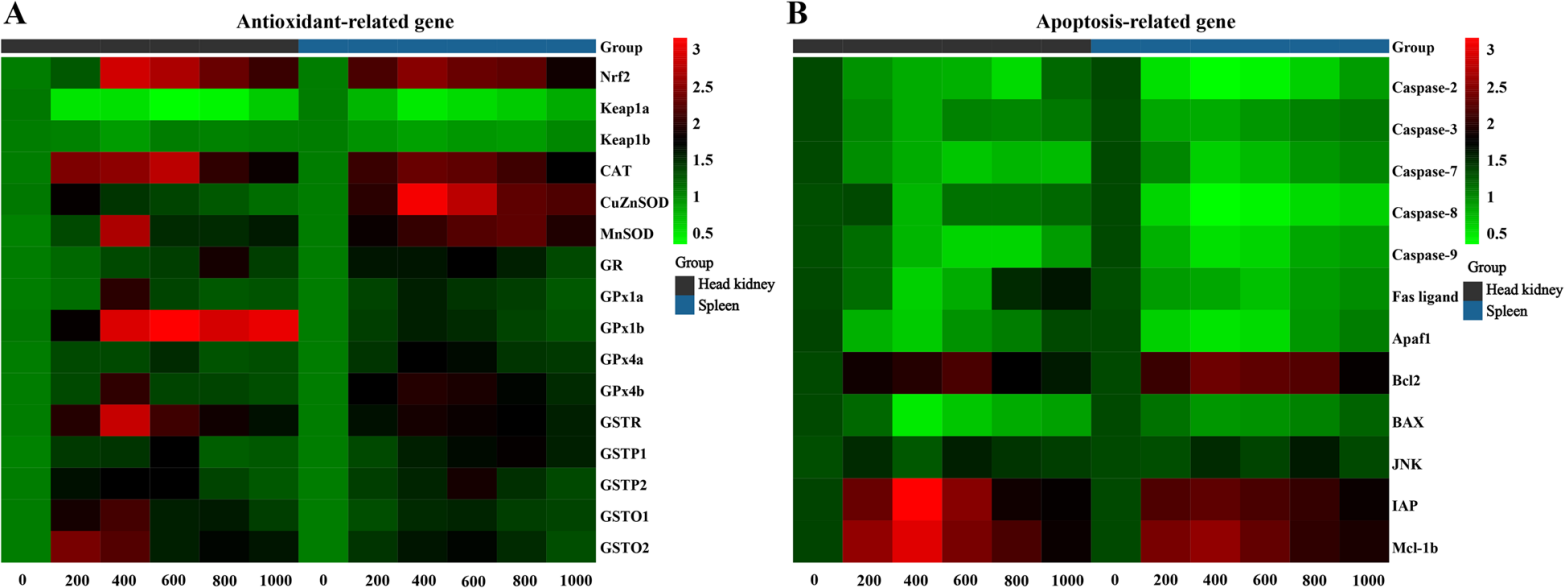
Heat-map of different levels of MOS changed expression of antioxidant-related parameters in the head kidney and spleen of grass carp after infection of *Aeromonas hydrophila*. The signal values of up-regulation (red) and down-regulation (blue) were expressed and ranged from 0.5 to 3.0 folds

As shown in Fig. 3A and B, the head kidney and spleen exhibited expression of genes related to the antioxidative activity. Compared with that in the control group, the maximum fold-change in the levels of the genes in head kidney was as follows: the mRNA levels of *GSTO2* (*P*_*quadratic*_ = 0.544, *F* = 0.751, *df* = 5) were the highest upon supplementation with 200 mg/kg MOS; the mRNA levels of *MnSOD* (*P*_*quadratic*_ = 0.512, *F* = 0.845, *df* = 5), *GPx1a* (*P*_*quadratic*_ = 0.489, *F* = 0.915, *df* = 5), *GPx4b* (*P*_*quadratic*_ = 0.377, *F* = 1.375, *df* = 5), *GSTR* (*P*_*quadratic*_ = 0.055, *F* = 4.258, *df* = 5), *GSTP2* (*P*_*quadratic*_ = 0.053, *F* = 9.132, *df* = 5), and *GSTO1* (*P*_*quadratic*_ = 0.241, *F* = 2.369, *df* = 5) were the highest upon supplementation with 400 mg/kg MOS; the mRNA levels of *CAT* (*P*_*quadratic*_ = 0.065, *F* = 7.782, *df* = 5), *GPx1b* (*P*_*quadratic*_ = 0.011, *F* = 28.806, *df* = 5), and *GSTP1* (*P*_*quadratic*_ = 0.609, *F* = 3.232, *df* = 5) were the highest upon supplementation with 600 mg/kg MOS; the mRNA levels of *GR* (*P*_*quadratic*_ = 0.238, *F* = 2.408, *df* = 5) were the highest upon supplementation with 800 mg/kg MOS. Notably, mRNA levels of all these genes showed a gradual decline after a further increase in MOS concentration in the feed. Further, *CuZnSOD* (*P*_*quadratic*_ = 0.401, *F* = 1.257, *df* = 5) and *GPx4a* (*P*_*quadratic*_ = 0.124, *F* = 4.523, *df* = 5) expression showed an increasing trend; however, they did not show a significant change upon MOS supplementation. In the spleen, the maximum fold-change in the levels of the genes was as follows: the mRNA levels of *CAT* (*P*_*quadratic*_ = 0.020, *F* = 18.834, *df* = 5), *CuZnSOD* (*P*_*quadratic*_ = 0.059, *F* = 8.404, *df* = 5), *GPx1a* (*P*_*quadratic*_ = 0.026, *F* = 15.678, *df* = 5), *GPx1b* (*P*_*quadratic*_ = 0.040, *F* = 11.324, *df* = 5), *GPx4a* (*P*_*quadratic*_ = 0.054, *F* = 9.037, *df* = 5), *GPx4b* (*P*_*quadratic*_ = 0.022, *F* = 17.729, *df* = 5), and *GSTR* (*P*_*quadratic*_ = 0.013, *F* = 25.875, *df* = 5) were the highest upon supplementation with 400 mg/kg MOS; the mRNA levels of *GR* (*P*_*quadrati c*_ = 0.025, *F* = 15.937, *df* = 5), *GSTP2* (*P*_*quadratic*_ = 0.059, *F* = 8.439, *df* = 5), *GSTO1* (*P*_*quadratic*_ = 0.013, *F* = 24.968, *df* = 5), and *GSTO2* (*P*_*quadratic*_ = 0.004, *F* = 53.704, *df* = 5) were the highest upon supplementation with 600 mg/kg MOS, the expression of *MnSOD* (*P*_*quadratic*_ = 0.003, *F* = 64.067, *df* = 5) and *GSTP1* (*P*_*quadratic*_ = 0.004, *F* = 59.752, *df* = 5) were the highest upon supplementation with 800 mg/kg MOS. In addition, antioxidant regulatory signaling molecules *Nrf2* (head kidney, *P*_*quadratic*_ = 0.099, *F* = 5.508, *df* = 5; spleen, *P*_*quadratic*_ = 0.020, *F* = 18.599, *df* = 5) and *keap1a* (head kidney, *P*_*quadratic*_ = 0.032, *F* = 13.502, *df* = 5; spleen, *P*_*quadratic*_ = 0.014, *F* = 23.962, *df* = 5) showed a significant quadratic effect in these two organs (*P* < 0.05), whereas *keap1b* (*P*_*quadratic*_ = 0.511, *F* = 0.847, *df* = 5) levels showed no significant change with MOS supplementation in the head kidney.

### Expression of apoptosis-related genes

**Fig. 4:**
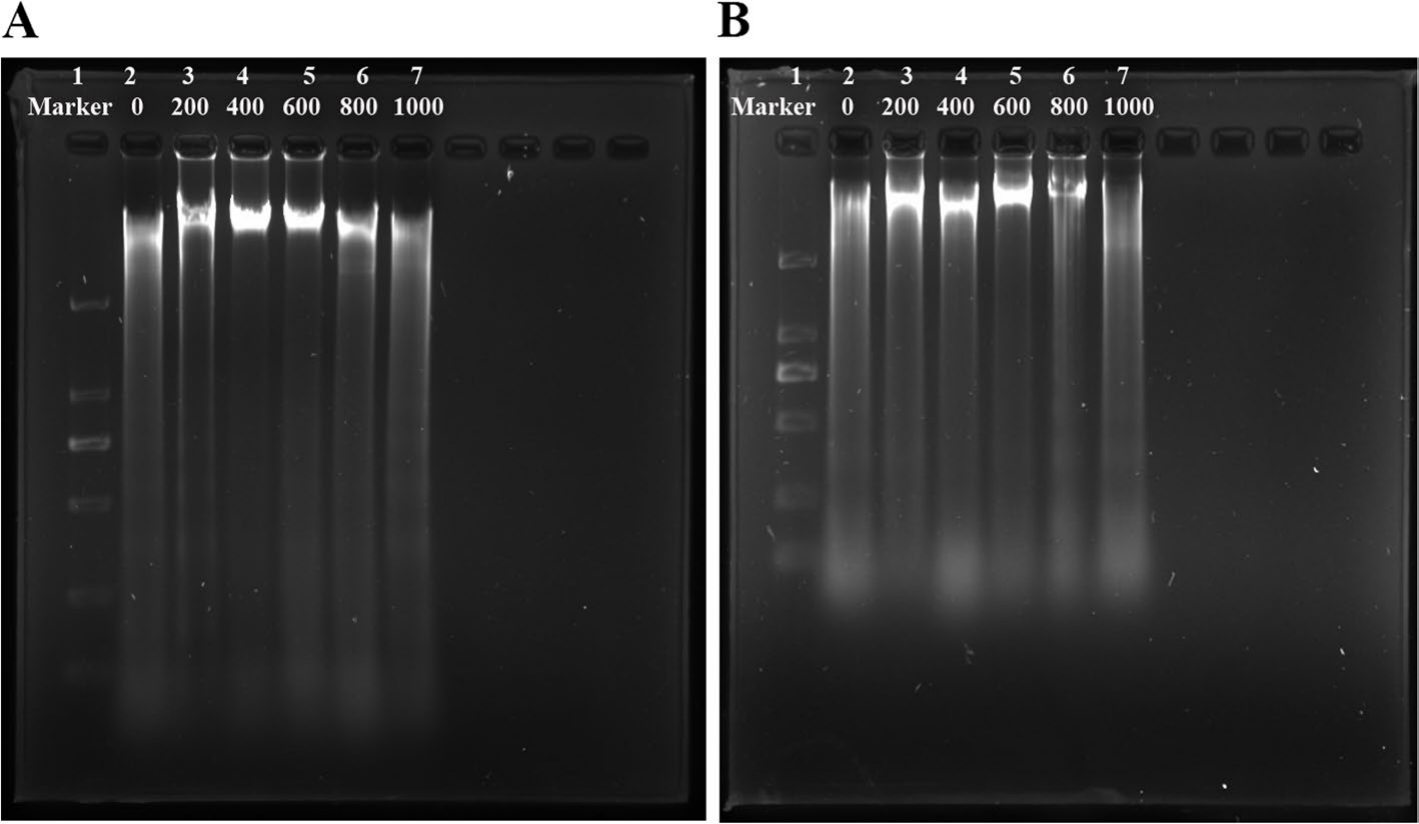
DNA fragmentation analysis in the head kidney (**A**) and spleen (**B**) of grass carp after infection of *Aeromonas hydrophila*. Lane 1: maker. Lane 2–7: levels of dietary MOS were 0, 200, 400, 600, 800 and 1,000 mg/kg, respectively. This experiment was repeated three times with similar results achieved

Upon infection with *A. hydrophila*, DNA isolated from the head kidney and spleen showed apparent fragmentation, as shown in Fig. 4A and B. The 600 and 800 mg/kg MOS-supplemented groups displayed reduced DNA fragmentation. A heatmap illustrating the expression of apoptosis-related genes in the head kidney and spleen is presented in Fig. 3A and B. Compared with that in the control group, in the head kidney, the maximum fold-change was as follows: the expression of Caspase-3 (*P*_*quadratic*_ = 0.136, *F* = 4.177, *df* = 5), Caspase-8 (*P*_*quadratic*_ = 0.481, *F* = 0.944, *df* = 5), *Fas* ligand (*P*_*quadratic*_ = 0.126, *F* = 4.473, *df* = 5), *Apaf1* (*P*_*quadratic*_ = 0.077, *F* = 6.753, *df* = 5), *BAX* (*P*_*quadratic*_ = 0.109, *F* = 5.057, *df* = 5), *IAP* (*P*_*quadratic*_ = 0.302, *F* = 3.821, *df* = 5) and *Mcl-1b* (*P*_*quadratic*_ = 0.100, *F* = 5.447, *df* = 5) was the lowest upon supplementation with 400 mg/kg, the expression of Caspase-7 (*P*_*quadratic*_ = 0.008, *F* = 34.968, *df* = 5), Caspase-9 (*P*_*quadratic*_ = 0.021, *F* = 18.396, *df* = 5), *Bcl2* (*P*_*quadratic*_ = 0.041, *F* = 11.026, *df* = 5), and *JNK* (*P*_*quadratic*_ = 0.79, *F* = 0.256, *df* = 5) was the lowest in the group supplemented with 600 mg/kg of MOS. Only Caspase-2 (*P*_*quadratic*_ = 0.087, *F* = 6.123, *df* = 5) levels were the lowest in the group supplemented with 800 mg/kg MOS. In the spleen, the mRNA levels of Caspase-2 (*P*_*quadratic*_ = 0.029, *F* = 14.574, *df* = 5), Caspase-3 (*P*_*quadratic*_ = 0.144, *F* = 3.948, *df* = 5), Caspase-7 (*P*_*quadratic*_ = 0.040, *F* = 11.380, *df* = 5), Caspase-8 (*P*_*quadratic*_ = 0.040, *F* = 11.367, *df* = 5), Caspase-9 (*P*_*quadratic*_ = 0.042, *F* = 10.893, *df* = 5), *Apaf1* (*P*_*quadratic*_ = 0.062, *F* = 8.088, *df* = 5), *Bcl2* (*P*_*quadratic*_ = 0.007, *F* = 38.045, *df* = 5), *BAX* (*P*_*quadratic*_ = 0.038, *F* = 79.07, *df* = 5), *IAP* (*P*_*quadratic*_ = 0.063, *F* = 7.984, *df* = 5), and *Mcl-1b* (*P*_*quadratic*_ = 0.121, *F* = 4.618, *df* = 5) were the lowest in the group supplemented with 400 mg/kg MOS. The mRNA levels of *Fas* ligand (*P*_*quadratic*_ = 0.024, *F* = 16.462, *df* = 5) were the lowest in the group supplemented with 600 mg/kg of MOS, and *JNK* (*P*_*quadratic*_ = 0.906, *F* = 0.727, *df* = 5) levels were the lowest in the group supplemented with 800 mg/kg MOS.

### Correlation analysis

**Fig. 5:**
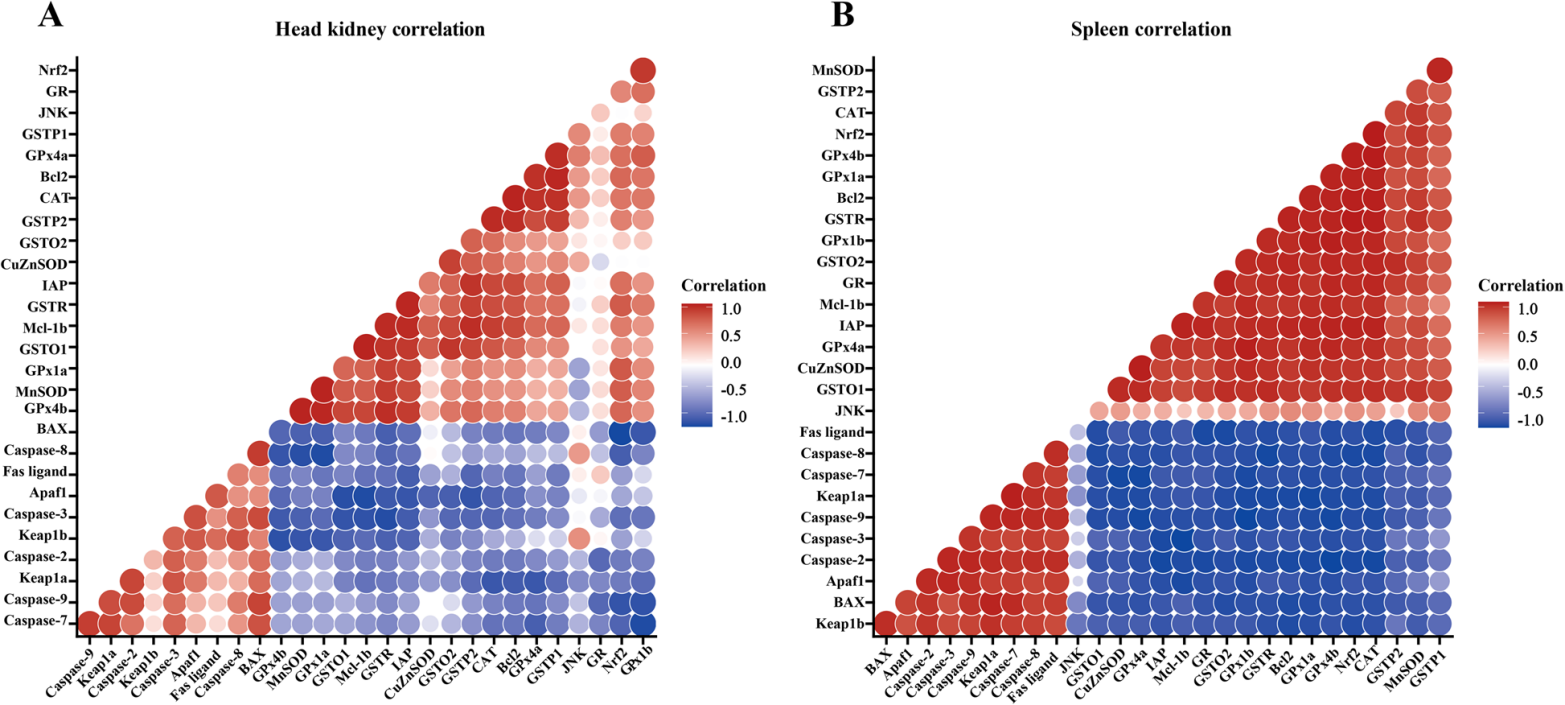
Correlation analysis of parameters in the head kidney and spleen of grass carp after infection of *Aeromonas hydrophila*. Head kidney (**A**) and spleen (**B**) of grass carp after infection of *Aeromonas hydrophila*

An illustration of the correlation analysis of gene expression is shown in Fig. 5A and B. Our data showed that expression of genes coding for antioxidant enzymes was positively correlated with *Nrf2* expression, whereas *Keap1a* and *Keap1b* expression were negatively correlated in the head kidney and spleen. In addition, the expression of genes for the pro-apoptotic factors (effector Caspases 3/7, initiator Caspases 8/9, and related signal molecules) of extrinsic and intrinsic apoptotic pathways were positively correlated with *Fas* ligand and *Apaf1*, whereas the expression of genes coding for anti-apoptotic factors (such as *Bcl-2*, *IAP* and *Mcl-1*) displayed a positive correlation with *Nrf2* expression in the head kidney and spleen.

### Key role and protein levels of Nrf2

**Fig. 6:**
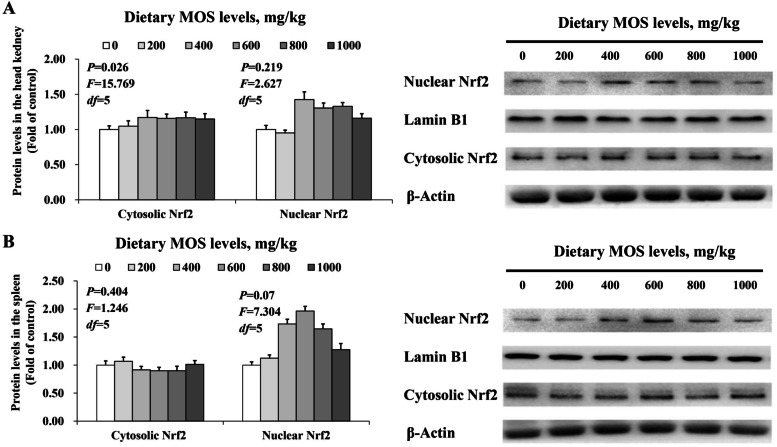
Western blot analysis of nuclear Nrf2 protein levels in the head kidney and spleen of grass carp after infection of *Aeromonas hydrophila*. **A**, head kidney; **B**, spleen. Data represent means of three fish in each group, error bars indicate SD. Values having different letters are significantly different

Figure 6A and B illustrates the results of an assay (Western blot) for cytosolic Nrf2 and nuclear Nrf2 protein expression in fish head kidneys and spleens, respectively. In comparison with the control group, in the head kidney, the nuclear Nrf2 levels (*P*_*quadratic*_ = 0.219, *F* = 2.627, *df* = 5) were highest with MOS supplementation up to 400 mg/kg, then plateaued (*P* < 0.05). In the spleen, the nuclear Nrf2 levels (*P*_*quadratic*_ = 0.070, *F* = 7.304, *df* = 5) were highest at the 0–600 mg/kg MOS supplementation and then plateaued.

## Discussion

We conducted the present in grass carp used in our previous work to investigate the protective effects of MOS supplementation on infected fish [18]. We previously reported that fish growth, as well as intestinal and skin health, can be improved by administering optimal quantities of MOS. Most researchers consider fish growth and development strongly correlated with intestinal barrier function [22, 41, 42]. Nevertheless, functional organs (head kidney and spleen) also play a crucial role in maintaining fish health [5, 6]. As previously demonstrated, MOS can enhance intestinal antioxidant capacity when confronted with *A. hydrophila* [18]. However, this evidence does not provide a sufficient understanding of the protective function of MOS in the head kidney and spleen. Therefore, to acquire further evidence of the protective effects of MOS in fish head kidney and spleen health, based on previous research, we conducted relevant experiments. We examined the effects of MOS on the fish antioxidant capacity and apoptosis of the head kidney and spleen and further revealed relevant molecular mechanisms. Accordingly, we studied the effect of MOS supplementation on the antioxidant capacity of the head kidney and spleen in grass carp.

### MOS attenuates oxidative stress

Cellular redox balance plays a vital role in the health of functional organs [43]. *A. hydrophila*, one of the most common pathogenic microorganisms associated with aquatic environments, can cause oxidative stress in fish [44]. A recent study highlighted that the infection pattern of *A. hydrophila* is characterized by the stimulation of robust host production of ROS, leading to oxidative damage [45]. MDA and PC are recognized as indicators of ROS-induced cell damage, which can be decreased by the antioxidant system [46, 47]. Our data showed that supplementation with an appropriate concentration of MOS inhibits excessive ROS production and decreases the concentration of MDA and PC. Superoxide anions and hydroxyl radicals are the main components of reactive oxygen species, which can cause oxidative damage to cells when present in excess [48, 49]. Anti-superoxide anion (ASA) and anti-hydroxyl radical (AHR) reflect the ability of tissues to inhibit superoxide anions and strong free radicals. Our results also confirmed that MOS increased the concentration of ASA and AHR (except in the spleen), which is consistent with the results of our previous study on the gut of the grass carp. These results indicate that MOS supplementation decreases oxidative stress in the head kidney and spleen of fish by inhibiting oxidative damage. In addition, MOS has hydroxyl groups capable of reacting with superoxide anions, hydrogen peroxide, and hydroxyl radicals [50]. MOS was demonstrated to increase free radical scavenging [39], suggesting that MOS is an excellent natural antioxidant with extensive free radical-scavenging properties. Excessive free radical scavenging capacity in fish could also be attributed to the primary antioxidant enzymes [20]. Consequently, we examined the antioxidant enzymes in the head kidney and spleen.

### MOS enhances antioxidant capacity

Enzymatic antioxidants include several antioxidant enzymes such as CAT, MnSOD, CuZnSOD, GR, GPx, and GST [20]. In addition to reducing superoxide radicals into hydrogen peroxide, SOD catalyzes the elimination of oxygen and water from superoxide radicals via CAT. GPx is also involved in the reduction of hydrogen peroxide. In contrast, GST catalyzes the conjugation of oxidants into GSH, an electron donor, and GR reduces oxidized glutathione to GSH. Results of the present study showed that the optimum concentration (400–800 mg/kg) of MOS enhanced the activities of various antioxidant enzymes, including CAT, MnSOD, CuZnSOD, GR, GPx, and GST, in these two organs. Further, the activities of antioxidant enzymes were enhanced in the head kidney and spleen of fish after dietary MOS supplementation. Generally, the activities of antioxidant enzymes were found to be highly correlated with their mRNA levels [51]. We also found that MOS supplementation upregulated the expression of antioxidant enzymes and related isoforms in the head kidney and spleen (except *CuZnSOD* and *GPx4a* in the head kidney). These results suggest that the enhanced antioxidant enzyme activity may be partly due to MOS-induced upregulation of their mRNA levels. Antioxidant enzyme genes are regulated by *Nrf2*, which acts as a critical factor in combating oxidative stress and is degenerated by *Keap1* in the nucleus [27, 52]. It has been demonstrated in mouse liver that Nrf2 protein levels in the nucleus can reflect Nrf2 nuclear translocation from the cytoplasm [53]. The present study showed that an optimal MOS dosage could evidently upregulate the expression of *Nrf2* and downregulate *keap1a* expression in the head kidney and spleen. Moreover, our data showed that the protein expression of nuclear Nrf2 was upregulated with supplementation of feed with optimal MOS concentration in the head kidney and spleen. Based on these findings, we propose that MOS supplementation activated *Nrf2* signaling pathway in the head kidney and spleen by promoting Nrf2 nuclear translocation. Notably, *Keap1b* expression in the head kidney and spleen was not affected by MOS supplementation. A possible reason for this phenomenon may be partially related to the absorption of phosphorus. MOS has been shown to promote phosphorus digestion in weaned piglets [28]. Coincidentally, a previous study from our laboratory demonstrated that phosphorus levels did not affect Keap1b in the head kidney and spleen of grass carp [29]. Hence, this evidence partially supports our hypothesis. Moreover, MN9D cells were found to apoptosis upon exposure to excessive oxidative damage [54]. Therefore, we next examined the effects of MOS supplementation on apoptosis in the head kidney and spleen of fish.

### MOS inhibition of excessive apoptosis

Apoptosis is a highly regulated physiological process that maintains homeostasis within organisms [55]. Apoptosis plays a crucial role in the normal development and homeostasis of organisms as well as in the pathogenesis of bacterial infections [56]. Bacterial infections are usually accompanied by oxidative damage, which can aggravate apoptosis [57]. Typically, apoptosis occurs in mammals through two pathways: the death receptor pathway (Fas ligand/Caspase-8) and the mitochondrial pathway (Bcl-2, Mcl-1b, and Bax)/Apaf-1/Caspase-9 [58–60]. Apoptosis is triggered by Caspase-3 activation, a critical apoptotic protein. Apoptosis-related proteins include Caspase-8 and Caspase-9 apoptotic promoters and Caspase-3 and Caspase-7 apoptotic effectors [61]. DNA fragmentation is a classic phenotypic indicator of apoptosis [62]. The present study showed that DNA fragmentation was reduced with appropriate MOS supplementation (400–600 mg/kg) compared to the control. Our results also showed that the mRNA levels of Caspase-3, Caspase-7, Caspase-8, and Caspase-9 were downregulated upon supplementation with 400–600 mg/kg MOS compared with those in the control. The death receptor pathway and the critical mitochondrial pathway signaling molecules (e.g., *Fas* ligand, *BAX,* and *Apaf1*) were downregulated with MOS supplementation. Moreover, the expression of anti-apoptotic factors (*Bcl-2* and *Mcl-1b*) significantly increased with MOS supplementation. These data indicate that MOS supplementation suppressed excess apoptosis by inhibiting the death receptor pathway and mitochondrial pathway process in the head kidney and spleen after infection with *A. hydrophila*.

### Differences in the effects of MOS on different organs

Compared to our previous studies on multi-function organs (e.g., intestine and skin), the results of the stress response of the head kidney and spleen in the current study revealed a noteworthy result [18, 39]. The levels of biomarkers of oxidative damage (ROS, PC, and MDA) and mRNA levels of multiple antioxidative enzymes in the head kidney and spleen exhibited an overall trend similar to that of the intestine and skin. Notably, the expression of *GPx* and *GST* in the head kidney was higher than that in the spleen, whereas the expression of *CuZnSOD* and *MnSOD* in the spleen was significantly higher than that in the head kidney. A possible explanation for this phenomenon relates to the structural differences among different organs and MOS characteristics. Fish head kidney contain numerous macrophages that produce excessive ROS and inflammatory cytokines when stimulated by pathogens [6]. As mentioned earlier, free radicals such as superoxide anions, hydrogen peroxide, and hydroxyl radicals can react with MOS owing to their hydroxyl groups [50]. Thus, MOS can remove excess free radicals from organisms. Therefore, feedback regulation likely occurs without requiring more SOD to participate in free radical scavenging. These results are consistent with biochemical indicators in the head kidney, showing that MOS did not affect CuZnSOD activity, supporting our hypothesis. In conclusion, we also consider that the effect of MOS supplementation on the antioxidant stress response of different organs in fish is determined by many factors, including feeding time, dosage, pathogen, and organ structure; however, further studies are necessary to elucidate this phenomenon.

## Conclusion

Combining the above data and discussion, we can conclude that adding prebiotics to diets improves the antioxidant capacity of fish and increases disease resistance in the head kidney and spleen, which is significant in modern intensive aquaculture. The present study improves our knowledge regarding using MOS as a prebiotic in freshwater fish diets. Our data indicated that dietary MOS supplementation could effectively alleviate oxidative stress to fish under pathogen-challenge conditions by enhancing antioxidant capacity and inhibiting excessive apoptosis in the head kidney and spleen. In addition, we further revealed that MOS enhanced antioxidant capacity by activating the Nrf2/Keap1 signaling pathway in the fish head kidney and spleen. Based on the levels of biomarkers (ROS, MDA, and PC) of oxidative damage in the head kidney and spleen, optimal MOS supplementation recommended is 575.21, 557.58, 531.86, 597.35, 570.16, and 553.80 mg/kg, respectively (Table 3). These findings demonstrate the critical role of prebiotics in mediating excessive oxidative stress induced by *A. hydrophila*. Our results contribute to the investigation of functional nutritional strategies to attenuate oxidative stress responses induced by pathogenic bacterial stress in aquaculture.

**Table 3:** The optimal MOS dosage based on different indices for grass carp

Indices	Regressive equation	*R*^2^	*F*-value	*P*_*Quadratic*_	MOS, mg/kg
Head kidney					
ROS	*y* = 112.15*x*^2 ^– 129.02*x* + 99.35	0.97	42.492	0.006	575.21
MDA	*y* = 4.95*x*^2 ^– 5.52*x* + 4.73	0.89	11.846	0.038	557.58
PC	*y* = 13.34*x*^2 ^– 14.19*x* + 8.56	0.76	4.824	0.116	531.86
Spleen					
ROS	*y* = 134.82*x*^2 ^– 161.07*x* + 97.92	0.97	57.812	0.004	597.35
MDA	*y* = 7.34*x*^2 ^– 8.37*x* + 9.86	0.86	9.108	0.053	570.16
PC	*y* = 4.74*x*^2 ^– 5.25*x* + 3.72	0.91	15.327	0.027	553.80

## Supplementary Information

**Additional file 1: Table S1.** Real-time PCR primer sequences.

**Additional file 2: Table S2.** The information of antibodies (Western blot).

## Data Availability

The datasets are included in this article and available from the corresponding author on reasonable request.

## Abbreviations

AHR: Anti-hydroxyl radical
Apaf-1: Apoptotic protease activating factor-1
ASA: Anti-superoxide anion
BAX: Bcl2 associated X protein
Bcl2: B-cell lymphoma protein-2
Caspase: Cysteinyl aspartic acid-protease
CAT: Catalase
CuZnSOD: Copper/zinc superoxide dismutase
Fas ligand: Fatty acid synthetase ligand
GAPDH: Glyceraldehyde-3-phosphate dehydrogenase
GPx: Glutathione peroxidase
GR: Glutathione reductase
GSH: Glutathione
GST: Glutathione-S-transferase
IAP: Inhibitor of apoptosis proteins
JNK: C-Jun N-terminal protein kinase
Keap1: Kelch-like-ECH-associated protein 1
Mcl-1b: Myeloid cell leukemia-1b
MDA: Malondialdehyde
MnSOD: manganese superoxide dismutase
MOS: Mannan oligosaccharides
Nrf2: NF-E2-related factor 2
PC: Protein carbonyl
ROS: Reactive oxygen species
