# Supplementary material for: Mannan oligosaccharides alleviate oxidative injury in the head kidney and spleen in grass carp (Ctenopharyngodon idella) via the Nrf2 signaling pathway after Aeromonas hydrophila infection
Source: J Anim Sci Biotechnol. 2023 Apr 15;14:58. doi: 10.1186/s40104-023-00844-1 (PMC10105433; doi:10.1186/s40104-023-00844-1)
Supplement: Supplementary file 1 — Additional file 1: Table S1. Real-time PCR primer sequences. [file 40104_2023_844_MOESM1_ESM.docx]

**Table S1** Real-time PCR primer sequences

| **Target gene^1^** | **Primer sequence (5’→3’)** | **Temperature, °C** | **Accession number** |
| --- | --- | --- | --- |
| *CuZnSOD* | Forward: CGCACTTCAACCCTTACA | 61.5 | GU901214 |
|  | Reverse: ACTTTCCTCATTGCCTCC |  |  |
| *MnSOD* | Forward: ACGACCCAAGTCTCCCTA | 60.4 | GU218534 |
|  | Reverse: ACCCTGTGGTTCTCCTCC |  |  |
| *CAT* | Forward: GAAGTTCTACACCGATGAGG | 58.7 | FJ560431 |
|  | Reverse: CCAGAAATCCCAAACCAT |  |  |
| *GPx1a* | Forward: GGGCTGGTTATTCTGGGC | 61.5 | EU828796 |
|  | Reverse: AGGCGATGTCATTCCTGTTC |  |  |
| *GPx1b* | Forward: TTTTGTCCTTGAAGTATGTCCGTC | 60.3 | KT757315 |
|  | Reverse: GGGTCGTTCATAAAGGGCATT |  |  |
| *GPx4a* | Forward: TACGCTGAGAGAGGTTTACACAT | 60.4 | KU255598 |
|  | Reverse: CTTTTCCATTGGGTTGTTCC |  |  |
| *GPx4b* | Forward: CTGGAGAAATACAGGGGTTACG | 60.3 | KU255599 |
|  | Reverse: CTCCTGCTTTCCGAACTGGT |  |  |
| *GSTR* | Forward: TCTCAAGGAACCCGTCTG | 58.4 | EU107283 |
|  | Reverse: CCAAGTATCCGTCCCACA |  |  |
| *GSTp1* | Forward: ACAGTTGCCCAAGTTCCAG | 59.3 | KM112099 |
|  | Reverse: CCTCACAGTCGTTTTTTCCA |  |  |
| *GSTp2* | Forward: TGCCTTGAAGATTATGCTGG | 59.3 | KP125490 |
|  | Reverse: GCTGGCTTTTATTTCACCCT |  |  |
| *GSTo1* | Forward: GGTGCTCAATGCCAAGGGAA | 58.4 | KT757314 |
|  | Reverse: CTCAAACGGGTCGGATGGAA |  |  |
| *GSTo2* | Forward: CTGCTCCCATCAGACCCATTT | 61.4 | KU245630 |
|  | Reverse: TCTCCCCTTTTCTTGCCCATA |  |  |
| *GR* | Forward: GTGTCCAACTTCTCCTGTG | 59.4 | JX854448 |
|  | Reverse: ACTCTGGGGTCCAAAACG |  |  |
| *Nrf2* | Forward: CTGGACGAGGAGACTGGA | 62.5 | KF733814 |
|  | Reverse: ATCTGTGGTAGGTGGAAC |  |  |
| *Keap1a* | Forward: TTCCACGCCCTCCTCAA | 63.0 | KF811013 |
|  | Reverse: TGTACCCTCCCGCTATG |  |  |
| *Keap1b* | Forward: TCTGCTGTATGCGGTGGGC | 57.9 | KJ729125 |
|  | Reverse: CTCCTCCATTCATCTTTCTCG |  |  |
| *Fas Ligand* | Forward: AGGAAATGCCCGCACAAATG | 61.4 | KT445873 |
|  | Reverse: AACCGCTTTCATTGACCTGGAG |  |  |
| *JNK* | Forward: ACAGCGTAGATGTGGGTGATT | 62.3 | KT757312 |
|  | Reverse: GCTCAAGGTTGTGGTCATACG |  |  |
| *Bcl-2* | Forward: AGGAAAATGGAGGTTGGGAT | 60.3 | JQ713862 |
|  | Reverse: CTGAGCAAAAAAGGCGATG |  |  |
| *Mcl-1b* | Forward: TGGAAAGTCTCGTGGTAAAGCA | 58.4 | KT757307 |
|  | Reverse: ATCGCTGAAGATTTCTGTTGCC |  |  |
| *Bax* | Forward: CATCTATGAGCGGGTTCGTC | 60.3 | JQ793788 |
|  | Reverse: TTTATGGCTGGGGTCACACA |  |  |
| *Apaf-1* | Forward: AAGTTCTGGAGCCTGGACAC | 61.4 | KM279717 |
|  | Reverse: AACTCAAGACCCCACAGCAC |  |  |
| *IAP* | Forward: CACAATCCTGGTATGCGTCG | 58.4 | FJ593503.1 |
|  | Reverse: GGGTAATGCCTCTGGTGCTC |  |  |
| *Caspase-2* | Forward: CGCTGTTGTGTGTTTACTGTCTCA | 60.3 | KT757313 |
|  | Reverse: ACGCCATTATCCATCTCCTCTC |  |  |
| *Caspase-3* | Forward: GCTGTGCTTCATTTGTTTG | 55.9 | JQ793789 |
|  | Reverse: TCTGAGATGTTATGGCTGTC |  |  |
| *Caspase-7* | Forward: GCCATTACAGGATTGTTTCACC | 57.1 | KT625601 |
|  | Reverse: CCTTATCTGTGCCATTGCGT |  |  |
| *Caspase-8* | Forward: ATCTGGTTGAAATCCGTGAA | 59.0 | KM016991 |
|  | Reverse: TCCATCTGATGCCCATACAC |  |  |
| *Caspase-9* | Forward: CTGTGGCGGAGGTGAGAA | 59.0 | JQ793787 |
|  | Reverse: GTGCTGGAGGACATGGGAAT |  |  |
| *GAPDH* | Forward: GTTACAAGGGAGAAGTTCACCAT | 58.0 | GQ266395 |
|  | Reverse: CCGGTAGACTCGACTACATACAG |  |  |
| *β-actin* | Forward: GGCTGTGCTGTCCCTGTA | 61.4 | M25013 |
|  | Reverse: GGGCATAACCCTCGTAGAT |  |  |

^1^ CuZnSOD, copper, zinc superoxide dismutase; MnSOD, manganese superoxide dismutase; CAT, catalase; GPx, glutathione peroxidase; GST, glutathione-S-transferase; GR, glutathione reductase; Nrf2, NF-E2-related factor 2; Keap1, Kelch-like-ECH-associated protein 1; GAPDH, glyceraldehyde-3-phosphate dehydrogenase. Fas Ligand, fatty acid synthetase ligand; JNK, c-Jun Nterminal protein kinase; Bcl2, B-cell lymphoma protein-2; Mcl-1b, myeloid cell leukemia-1; Bax, Bcl-2 associated X protein; Apaf-1, apoptotic protease activating factor-1; IAP, inhibitor ofapoptosis proteins; Caspase, cysteinyl aspartic acid-protease.
