# Supplementary material for: Mannan oligosaccharides alleviate oxidative injury in the head kidney and spleen in grass carp (Ctenopharyngodon idella) via the Nrf2 signaling pathway after Aeromonas hydrophila infection
Source: J Anim Sci Biotechnol. 2023 Apr 15;14:58. doi: 10.1186/s40104-023-00844-1 (PMC10105433; doi:10.1186/s40104-023-00844-1)
Supplement: Supplementary file 2 — Additional file 2: Table S2. The information of antibodies (Western blot). [file 40104_2023_844_MOESM2_ESM.docx]

**Table S2** The information of antibodies (Western blot)

| **Indices** | **Host** | **Source** | **Catalog No.** | **Dilution for WB** |
| --- | --- | --- | --- | --- |
| Nucleus Nrf2 | Rabbit | Abcam (Cambridge, MA, USA) | ab31163 | 1:1,000 |
| β-actin | Rabbit | Affinity (Golden, Colorado, USA) | AF7018 | 1:3,000 |
| LaminB1 | Rabbit | Affinity (Golden, Colorado, USA) | AF5161 | 1:1,000 |
